# Fairness and Tax Morale in Developing Countries

**DOI:** 10.1007/s12116-023-09394-z

**Published:** 2023-03-31

**Authors:** Néstor Castañeda

**Affiliations:** Institute of the Americas, University College London, London, UK

**Keywords:** Tax morale, Fairness, Preferences for redistribution, Tax compliance

## Abstract

This paper investigates the relationship between individuals' attitudes towards fairness and their views about tax compliance in developing countries. It argues that individuals’ attitudes regarding fairness shape their views about paying taxes and their ethical stances regarding tax evasion. Using survey data for 18 major cities in Latin America, we find that individuals who are highly sensitive to fairness are less likely to consider paying taxes as a civic duty and more likely to justify tax evasion. These attitudes toward tax compliance are not inelastic. We also find evidence that individualst argues about reciprocity and merit mediate the effect of fairness on personal views about tax compliance. Finally, this paper shows that the heuristics people use to explain their position in the income distribution make them sensitive to inequality, and it affects their tax morale. These findings help us better understand the concept of reciprocity and provide valuable lessons on the urgent task of expanding fiscal capacity to promote economic growth and inequality in developing countries.

## Introduction

Low-income and developing countries typically collect fewer taxes than high-income countries (Besley and Persson 2014). Their tax structures are also less progressive, more dependent on indirect and trade taxes, and social security contributions are lower (Besley and Persson 2014). Developing countries are also the most affected by tax evasion, depriving them of substantial public revenue yearly (IMF 2015). A large body of research has explored how institutional factors and special interest politics reduce tax capacity in developing countries (Besley and Persson 2014; Flores-Macias 2019). For example, scholars have shown that “the combination of an informal economic structure, income from natural resources or specific commodities, and the availability of aid (for some countries) push many low-income countries into a situation of low tax/GDP ratios levied on a narrow tax base and a narrow set of individuals” (Besley and Persson 2014, p. 112).

In these contexts, special interest groups are also remarkably successful in shaping tax policy, reducing tax liabilities, and transferring taxation costs to poorly organized groups of citizens or the middle class (Fairfield 2015; Castañeda 2017; Castañeda and Doyle 2019). These structural factors put additional pressure on the already narrow tax bases by lowering taxpayers’ morale and opening the door to widespread tax evasion. Therefore, understanding tax compliance’s behavioral determinants is critical to find ways to expand tax bases and boost tax revenues in emergent economies.

Behavioral economics literature shows that deterrence mechanisms, individual-level intrinsic motivations (or beliefs), and social norms are important determinants of tax compliance (Allingham and Sandmo 1972; Fellner et al. 2013; Luttmer and Singhal 2014; Pickhardt and Prinz 2014; Castro and Scartascini 2015; Dwenger et al. 2016; Kettle et al. 2016; Slemrod 2017, 2018; Alm 2019). Moreover, there is strong evidence that individuals might comply when their cost–benefit calculations favor paying taxes—tax compliance increases with higher audit probabilities and more severe fines (Allingham and Sandmo 1972; Feld and Larsen 2012; Alm et al. 2012; Slemrod 2018; Alm 2019). Still, some scholars have shown that “non-pecuniary motivations” (Luttmer and Singhal 2014, 150) like pride, positive self-image, honesty, or the fulfillment of civic duties are essential to understand taxpayers’ decisions (Luttmer and Singhal 2014; Dwenger et al. 2016; Bergolo et al. 2020). There is also evidence that people’s desire to conform to the behavior of peers (Benabou and Tirole 2011; Del Carpio 2014; Castro and Scartascini 2015; Alm et al. 2017) and persistent social norms also shape tax compliance (Cummings et al. 2009; Benabou and Tirole 2011; DeBacker et al. 2015; Lefebvre et al. 2015; Hallsworth et al. 2017). Information issues and the complexity of tax incentives also seem to shape tax compliance (Hashimzade et al. 2013; Abeler and Jäger 2015; Bhargava and Manoli 2015; Castro and Scartascini 2015; Perez-Truglia and Troiano 2018).

While much has been said about the individual motivations for tax compliance, political economy literature still pays little attention to the influence of individual attitudes toward fairness and redistribution on tax morale. Some scholars have shown that reciprocity—or the idea that individuals’ willingness to pay taxes is conditional on their views about public goods provision— affects tax morale. They have found solid evidence that individuals are indeed more willing to pay their taxes when they trust their governments, are satisfied with the delivery of public goods, or receive reciprocal benefits in return for paying taxes (Torgler 2003, 2005; Daude et al. 2013; Kuziemko et al. 2015; Castro and Scartascini 2015; Ortega et al. 2016; Ballard-Rosa et al. 2017; Castañeda et al. 2020; Carrillo et al. 2021).

However, the effect of individual attitudes toward inequality and fairness on tax compliance has received much less attention. Based on the notion of quasi-voluntary compliance (Levi 1989), some scholars have studied how willingness to pay taxes is conditioned upon the degree to which individuals perceive public goods provision to be fair or the tax structure to be progressive (Cummings et al. 2009; Doerrenberg and Peichl 2013; Heinemann and Kocher 2013; Beramendi and Rehm 2016; Ballard-Rosa et al. 2017; Castañeda et al. 2020). Other scholars argue that taxpayers are more inclined to evade taxes if they view themselves unfairly treated by the tax system (Bordignon 1993; Fortin et al. 2007; Spicer and Becker 1980), and this effect seems to be conditional on their income and working conditions (Barth et al. 2013). In any case, this literature shows us that people also make altruistic considerations when evaluating tax policies. Thus, one can reasonably argue that attitudes toward inequality and social fairness matter for tax compliance and that more progressive tax schedules could boost tax morale.

This article investigates whether people’s views on fairness shape tax morale in highly unequal contexts. I show that individuals who think the tax structure is unfair (i.e., it favors rich people) are less likely to consider that paying taxes is a trait of good citizens and more likely to justify tax evasion. Moreover, this article shows that unfavorable views about fairness (e.g., when individuals believe that taxes on rich people are too low) significantly reduce morale among taxpayers. Similarly, I show that individuals who believe luck is more important than hard work for personal economic success are less likely to think that paying taxes is a trait of good citizens and more likely to justify tax evasion. Consequently, this paper presents evidence that the relationship between attitudes toward fairness and tax evasion critically hinges on individual redistribution preferences. In other words, more progressive tax preferences lead to higher tax morale.

This article contributes to the tax compliance literature by analyzing the often-neglected relationship between attitudes toward fairness and tax morale. It also contributes to the extensive literature on tax morale, but it mainly advances our understanding of it in highly unequal countries. In particular, we use the literature on tax morale in developed countries as a starting point to show that the nature of this linkage is different in contexts where the levels of inequality are high because high exposure to inequality could make people more (or less) sensitive to changes in the distribution of income or wealth and the government action to reduce inequities. Like in similar studies for developed countries, I find a positive relationship between fairness and tax morale. However, in highly unequal contexts, this relationship is significantly mediated by people’s assessment of the provision of public goods. Therefore, this paper presents evidence from Latin America that expands our understanding of the mechanisms that make reciprocity consequential for tax morale. Finally, this article contributes to the literature on tax capacity in developing countries by showing that the behavioral consequences of persistent inequality prevent already weak governments from increasing their revenues and expanding their fiscal space.

I organize the article as follows. First, I review the related literature and present my working hypotheses. Then, I describe the data and statistical models used for the empirical analysis. Next, I report the main findings of this study and present the results of some robustness tests. Finally, I discuss the implications of these findings for studying tax compliance in developing economies.

## Attitudes Toward Fairness and Tax Morale

There is plenty of literature analyzing the effect of inequality on demand for redistribution. However, our understanding of the link between individual perceptions of fairness and tax compliance is still limited. Recent studies offer empirical evidence that attitudes toward inequality affect individuals’ stances about tax progressivity (Doerrenberg and Peichl 2013; Beramendi and Rehm 2016; Ballard-Rosa et al. 2017; Alvarado 2018; Boudreau and MacKenzie 2018). This literature offers new ways to understand how individuals’ beliefs about inequality shape their beliefs about taxation and their support for progressive tax structures (Piketty 1995; Alesina and Angeletos 2005; Chow and Galak 2012; Durante et al. 2014; Agranov and Palfrey 2015; Lefgren et al. 2016; Lü and Scheve 2016; McCall et al. 2017; Sands 2017). However, we know relatively little about the impact of individuals’ views about fairness (in the form of either progressive tax treatment or compensation mechanisms) on tax morale. Is tax morale higher when individuals think that the tax structure is progressive? Do individuals find tax evasion justifiable when they have unfavorable views about fairness? Do these mechanisms work to the same extent in highly unequal societies?

To answer these questions, we need to establish a clear link between reciprocity and fairness considerations. Tax morale literature has shown that, besides deterrence mechanisms (Allingham and Sandmo 1972; Kirchler 2007; Alm 2019), the social and institutional environment in which individuals interact with each other could significantly affect their tax morale (Luttmer and Singhal 2014; Alm 2019; Bergolo et al. 2020, p. 366). The notion of reciprocity (i.e., the idea that “willingness to pay taxes depends on the individual’s relationship with the state other than direct tax-benefit linkages” (Luttmer and Singhal 2014, p. 157)) encapsulates part of these environmental factors.

The literature on reciprocity shows that people’s tax morale will boost when they have positive views about the legitimacy of the state or the quality of government action because they directly benefit from the provision of public goods (Levi 1989; Scholz and Lubell 1998; Alm et al. 1993; Feld and Frey 2002; Frey and Meier 2004; Timmons 2005; Frey and Torgler 2007; Cummings et al. 2009; Feld and Larsen 2012; Luttmer and Singhal 2014; Bodea and LeBas 2016; Doerrenberg and Peichl 2018; Cullen et al. 2018; Castañeda et al. 2020; Carrillo et al. 2021). Therefore, citizens would be more willing to pay taxes if they are satisfied with public goods, trust their governments, and have no reliable private substitutes for public goods (Bodea and LeBas 2016; Castañeda et al. 2020).

However, reciprocity also involves other attributes of the relationship between individuals and the state—the so-called *fiscal contract* (Timmons 2005; Timmons and Garfias 2015; Oliver 2019; Besley 2020). For example, fairness considerations shape individuals’ views about reciprocity. When individuals form their opinions about the legitimacy of the social contract, they not only make self-interest considerations. They also use available information about inequality—e.g., their perceived position within the income distribution—to assess policy outcomes and the effectiveness of government action (Cruces et al. 2013). Therefore, rising inequality or the lack of government attention to increasing inequality could damage citizens’ reciprocity considerations, even among individuals who directly benefit from public goods. In other words, “people are motivated by forces other than self-interest, and particularly so by fairness considerations” (Isaksson and Lindskog 2009, p. 884).

If one understands taxation as a contract between citizens and the state, the prevalence of this contract would depend on (i) the incentives provided by the government to induce higher levels of effort among taxpayers via public spending and (ii) how taxpayers assess government actions to improve public goods and reduce inequality (Fehr and Gächter 2000). We could then expect that individuals are less willing to pay taxes (or more prone to evade taxes) in contexts where they perceive income inequality as rising or social mobility as stalling. People will also be less willing to pay taxes in contexts where governments do little to reduce inequality.

People may also be more sensitive to fairness considerations if they believe that circumstances beyond their control determine their financial conditions. For example, their beliefs about the causes of individual financial success or their experiences with social mobility (Piketty 1995; Benabou and Tirole 2011) may affect their views about fairness and, consequently, their opinions about reciprocity. Arguably, these experiences also shape people’s tax morale.

In this paper, I argue that when people evaluate their relationship with the state, they mainly focus on two facets of the fiscal exchange: their access to public goods and the degree of fairness. In other words, they make both selfish and altruistic considerations. Furthermore, I hypothesize that individuals’ beliefs about fairness determine whether they are willing to pay taxes. In addition to self-interest, people’s preferences are informed by equity and reciprocity concerns (Fong 2001; Alesina and Angeletos 2005; Cavaillé and Trump 2014; Scheve and Stasavage 2016, p. 42):

- H1: Individuals are less willing to pay taxes (or more prone to evading taxes) in contexts where they perceive inequality is rising.

Several scholars have recently explored this line of inquiry in the case of developed economies. For example, Cavaillé and Trump argue that “redistributive attitudes are not unidimensional: support for the redistribution of income by the government (‘redistribution from’) is empirically distinct from support for policies that help the poor and the unemployed (‘redistribution to’)” (Cavaillé and Trump 2014, p. 148). Cavaillé and Trump (2014) also argue that support for redistribution to the poor depends on social affinity and empathy; meanwhile, support for redistribution from the rich seems to be informed by individuals’ position as potential beneficiaries of redistribution. However, as recently argued by some scholars (Kuziemko et al. 2015; Beramendi et al. 2019; Stantcheva 2020), it is still unclear how people’s beliefs about redistribution translate into specific preferences and attitudes toward taxation. Thus, to disentangle how subjective perceptions (or misperceptions) about inequality or social fairness affect people’s attitudes toward taxes, we need to focus on specific aspects of these beliefs that are key to explaining their views on the tax structure.

In contrast to other facets of people’s beliefs about inequality like deservingness or social affinity—that are more relevant to explain people’s preferences for redistribution via social spending (e.g., Lupu and Pontusson 2011; Cavaillé and Trump 2014)—fairness considerations are critical to understanding people’s support for redistribution via taxation (Davidai 2018; Trump 2018; Sands and de Kadt 2020; Stantcheva 2020). Recent studies show that exposure to inequality increases people’s willingness to support heavier taxation on the rich (Sands 2017; Sands and de Kadt 2020). These studies also show that the experience of inequality seems to shape people’s redistributive preferences and their support for more progressive tax structures.

However, the link between perceived levels of inequality and people’s preferences for redistribution is indirect (Kuziemko et al. 2015; Stantcheva 2020). Indeed, recent studies suggest that factors such as individuals’ socioeconomic attributes or opinions about social mobility moderate the effect that perceived levels of inequality have on their preferences for redistribution (Brown-Iannuzzi et al. 2017; Condon and Wichowsky 2020). Fairness is only salient when people’s position in the income distribution and the heuristics they use to explain it make them particularly sensitive to inequality. In other words, individuals’ views about fairness affect tax morale, especially when those views make them aware of the drawback of inequality. Therefore, we can expect that:

The effect of fairness considerations on people's tax morale will vary according to:

- H2a: their attitudes toward reciprocity
- H2b: their o beliefs about the role of effort (or luck) in economic success.

Hypotheses H1, H2a, and H2b contend that self-interest and altruistic considerations explain tax morale. However, individuals are neither purely selfish subjects nor purely fair-minded subjects. This duality of purpose explains why unfair outcomes lower tax morale. In other words, fair-minded actors often behave as if they are strictly self-interested or vice versa (Fehr and Gächter 2000). Therefore, it seems crucial to understand under which circumstances self-interest or altruistic considerations become the most prevalent factor explaining people's views on tax compliance.

Understanding the “fairness side” of reciprocity is critical to the literature on the micro-foundations of tax and transfer systems in developing countries. As correctly argued by several scholars, the characteristics of the labor market (e.g., the size of informality), the recent evolution of the welfare system, or the scope of redistribution shape people’s expectations about the fiscal contract (Timmons 2005; Carnes and Mares 2014; Timmons and Garfias 2015; Holland and Schneider 2017; Holland 2018; Berens 2020). Holland (2018), for example, presents suggestive evidence that poor people in Latin America have diminished expectations about what the state can provide and redistribute. These diminished expectations could explain the region’s low electoral support for sharper redistribution policies (Holland and Schneider 2017). From this perspective, high levels of informality, regressive fiscal systems, and informal access barriers diminish poor people’s expectations about the fiscal contract in developing countries and make them less supportive of redistribution (Holland 2018).

In this paper, I argue that the experience of injustice also feeds people’s views on fairness and negatively affects their willingness to pay taxes. Proving the existence of a link between people’s views on fairness and their demand for redistribution goes beyond the scope of this article. However, we acknowledge that both factors are crucial to understanding the effect of reciprocity on tax morale.

## Data and Empirical Strategy

### Why Latin America?

Latin America is a perfect case to study the relationship between attitudes toward fairness and tax morale. Like in other developing regions, right and left governments have recently increased spending on social programs (Garay 2016). This expansion has successfully reduced poverty and inequality (López-Calva and Lustig 2010; Holland and Schneider 2017; ECLAC 2018). Poverty rates were reduced from 45.5% in 2002 to 30.8% in 2019 (ECLAC 2019, p. 97). The average Gini coefficient in the region decreased from 0.54 in 2002 to 0.46 in 2018 (ECLAC 2019, p. 42). Despite these improvements, Latin America remains one of the more unequal regions of the world. The regional Gini Index is still more than ten percentage points higher than the OECD average. The wealthiest 20% of the population holds about 11 times the income of the poorest 20% (ECLAC 2019). The average regional Gini coefficient for wealth inequality remains the highest globally (Jiménez 2015; ECLAC 2018). The economic impact of the COVID-19 pandemic still needs to be measured appropriately. However, preliminary assessments show that poverty and inequality have substantially increased due to the public health measures implemented to contain the advance of the virus in the region.

Persistent inequality in Latin America results from complex historical, political, and economic processes (Coatsworth 2008; López-Calva and Lustig 2010; Cornia 2014; Williamson 2015; ECLAC 2018; Sánchez-Ancochea 2020). However, fiscal policies undoubtedly help inequality perpetuate (Jiménez 2015; ECLAC 2018; Sánchez-Ancochea 2020). While in OECD countries, taxes reduce the Gini coefficient by almost 16 percentage points, in Latin America, that reduction is less than three percentage points (ECLAC 2018).

On the one hand, the total tax burden and the share of direct taxes as a percentage of total revenues are still very low regarding the region’s level of economic development (ECLAC 2018). The tax burden on household incomes only accounts for 1.4% of the GDP in Latin America. In contrast, personal income taxes are about 8.4% of the GDP in OECD countries and 10% of the GDP in the European Union (ECLAC 2018, pp. 87–88). On the other hand, tax evasion rates are high—e.g., the average income tax evasion rate is almost 50%—and tax avoidance is common among wealthy individuals and firms. Both low tax burdens and low levels of tax morale make governments highly dependent on regressive tax structures and prevent them from using taxes as effective redistribution tools (ECLAC 2018, pp. 88–91).

Why are taxes not effectively used as redistribution tools? On the one hand, empirical evidence suggests that governments do not promote fairer tax structures because business interest groups effectively use their political resources to restrain progressive taxation. There is now extensive literature (see Flores-Macias 2019) showing that business preferences prevail over collective interests because the business community is both structurally and instrumentally powerful (Fairfield 2015) or simply because they are better organized for collective action (Castañeda 2017, 2021; Castañeda and Doyle 2019). Unsurprisingly, indirect taxation seems higher where business interest groups are highly coordinated (Flores-Macías 2014; Castañeda 2017, 2021; Castañeda and Doyle 2019). The degree of coordination among business interest groups and the success of economic elites in obstructing tax-oriented redistribution explains the historical roots of regressive taxation in Latin America (Castañeda 2017, 2021; Castañeda and Doyle 2019; Ondetti 2015, 2017, 2021).

On the other hand, labor informality restricts governments’ ability to promote tax progressivity because many workers are effectively excluded from social protection systems, and it is difficult to catch them in payroll or income tax nets (Castañeda and Doyle 2019; Berens 2020). Also, access to social insurance is expensive for informal workers, and the coverage of non-contributory social insurance programs remains limited (Mesa-Lago 2008; Franzoni and Sánchez-Ancochea 2016; Cruz-Martínez 2019). The high degree of fragmentation of the labor market also creates strong incentives for individuals to opt out of fragile public welfare systems (Berens 2020). There is also evidence that differences in employment status affect policy preferences (Carnes and Mares 2014), constrain their electoral demands for redistribution (Holland 2018), and ultimately shape public attitudes toward progressive taxation (Berens and Gelepithis 2018).

Finally, politicians and policymakers have few electoral incentives to promote tax fairness. On the one hand, the chain of representation is malfunctioning, and there is a severe disconnection between citizens and politicians (Crisp et al. 2020). On the other hand, tax equity is not a salient issue for voters. Politicians do not promote more progressive tax structures because the public does not strongly demand tax fairness. Why do people not ask for tax fairness in highly unequal Latin America? We need to learn more about how exposure to inequality and fairness concerns shapes the preferences for redistribution of individuals in the region. It is still being determined under which circumstances citizens in the region are more willing to soak the rich and equalize the tax structure (Schwartz et al. 2023). It seems, as correctly suggested by Holland (2018), like the truncated nature of the social protection system makes the preferences of the poor and the rich look very similar, and politicians respond to those incentives. For the reasons presented above, Latin America is a perfect laboratory to investigate the individual-level determinants of tax morale.

### Data and Methods

In order to test the main argument of this paper, I estimate hierarchical and fixed-effects models using data provided by a large-scale regional survey conducted by the *Corporación Andina de Fomento* (*CAF*)-*Development Bank of Latin America* (CAF 2012) and one of the most recent waves of the *World Values Survey* (Inglehart et al. 2014). The *CAF Survey* is unique and examines citizen views on tax policy across 17 cities in 9 Latin American countries. The questionnaire includes several items to evaluate people’s attitudes toward taxation, their preferences for redistribution, and their assessment of public goods provision. To assess the external validity of the results, I have also estimated similar models using data provided by the *World Values Survey* (Inglehart et al. 2014).

For the dependent variable, I use two survey items that evaluate individuals’ attitudes toward tax evasion—i.e., their tax morale. The first item examines whether individuals consider paying taxes a civic duty. This item has values from 1 [never] to 10 [always]. The second item evaluates individuals' views on tax evasion. In this case, the survey asks respondents how justifiable it is to evade taxes on a scale of 1 [totally justifiable] to 10 [totally unjustifiable].

Figure 3 in the Appendix illustrates the density distributions of both variables. It shows substantial variation across categories but also evident skewness toward the higher point of the scale. Figure 4 in the Appendix shows that the mean values for both items vary across different units of analysis (cities). For example, the mean value of paying taxes as a civic duty is relatively high in Cordoba (Argentina) and Montevideo (Uruguay). In contrast, the mean values of this variable are pretty low in Sao Paulo (Brazil), Medellín (Colombia), and Caracas (Venezuela).

This article assesses whether individuals’ attitudes toward fairness and redistribution shape their tax morale. Measuring individual perceptions of fairness is a challenging task. People may have different perceptions of the levels of fairness and justice when they are asked to describe their societies. They may also have various informational paths to build those perceptions. Their political views or current social positions might shape these informational shortcuts. For example, recent scholarship has demonstrated that people’s views on fairness strongly correlate with current social status and opinions about social mobility mechanisms (Cruces et al. 2013; Hvidberg et al. 2020; Fehr et al. 2022).

Consequently, this paper uses three questions from the World Values Survey to investigate the public’s views on fairness in Latin America. First, I assess whether respondents think taxes on rich people are too low (do you think rich people pay [too little (1)—too much (10)] taxes?). This question seeks to directly assess people’s views on the fairness of the tax system. Second, I evaluate whether they believe that merit (i.e., hard work) is more important than luck for personal economic success (do you think hard work secures economic progress and social mobility?). In this case, I seek to evaluate whether people’s views on social mobility mechanisms affect their perception of fairness and, consequently, their tax morale. Finally, I measure the intensity of their preferences for redistribution by establishing whether they believe redistribution should be the top priority for the government (do you think reducing poverty and inequality should be the number one priority for the government?). In this case, I want to establish if egalitarian views on the role of the government could drive people’s opinions on taxation.

The theory section argues that people’s attitudes toward the social (fiscal) contract also shape their tax morale. To test this hypothesis, I assess whether or not reciprocity matters for the respondents in the sample. I evaluate whether or not individuals believe the government efficiently uses revenues to improve public utilities [(1) not agree to (10) strongly agree].

I also assess the impact of deterrence mechanisms. Conventional literature on tax evasion, primarily based on Allingham and Sandmo (1972)’s work, argues that citizens face a trade-off between paying taxes or cheating on their taxes, given existing audit-detection costs. From this perspective, tax evasion is more common if the probability of being caught and the costs of being sanctioned are low. To test this hypothesis, I measure the credibility of deterrence mechanisms by using a survey question that examines whether respondents consider legal sanctions for tax evasion lenient or severe—on a scale from 1 to 10.

Finally, I evaluate the impact of peer effects. Recent research on tax compliance demonstrates that peer effects significantly impact individual behavior (Luttmer and Singhal 2014). Indeed, this literature has examined factors such as group norms, bandwagon effects, conformity, social influence, or obedience to authority as crucial determinants of tax morale. The *CAF Survey* asks the respondents to roughly calculate the level of compliance among firms and individuals (how many firms/individuals pay their taxes? [none to all]). In this study, I measure the impact of peer effects by using the question on the perceived level of compliance among fellow citizens—how many fellow nationals do you believe pay taxes—from none (1) to all (10)?

In all model specifications, I control for the effect of relevant socioeconomic attributes such as gender, age, education, or participation in the labor market. Literature on tax morale has found strong empirical evidence that individual socioeconomic characteristics can shape individuals’ economic decisions and willingness to pay taxes (Luttmer and Singhal 2014). In this case, I also rely on data provided by the *CAF Survey*.

The data provided by the *CAF Survey* consist of individual responses nested within cities. Therefore, I estimate a series of variance-component, multilevel models to calculate the average deviation at the city level of the hierarchy. Based on these hierarchical models, I also calculate adjusted predictions for some of the most relevant independent variables to illustrate how these variables affect the probability that individuals are willing to evade taxes. Finally, I use the same technique to explain the interaction effects between fairness, reciprocity, and merit, which is crucial evidence to support my argument about the relationship between fairness and tax morale.

The models presented in the following section show preliminary evidence of the link between fairness and tax morale; however, they do not provide evidence of causal relations between these variables. I am aware of the potential endogeneity problems in the regression models and the need to use traditional approaches (e.g., instrumental variables) or experimental designs to deal with it. Therefore, I run basic statistical tests to detect multicollinearity or reverse causality problems. The results of these tests are not conclusive, but they suggest that endogeneity biases are not critical. In any case, the goal of this paper is more modest. I only want to illustrate the importance of the link between altruistic considerations and tax morale to understand the complexity of the concept of reciprocity.

## Findings

Table 1 shows the results of a mixed model specification using two different measures of tax morale to provide evidence for the main argument. Model (1) presents a hierarchical model estimating the correlation between individuals’ attitudes toward fairness and redistribution and their views about paying taxes as a civic duty when controlling for attitudes toward the social contract and essential socioeconomic attributes. Model (2) presents a hierarchical model estimating the correlation between individuals’ attitudes toward fairness and redistribution and their views about tax evasion when controlling for their attitudes toward the social contract and essential socioeconomic attributes. I estimate linear mixed-effects models with varying intercept group effects in both cases. As explained above, respondents are nested in cities—unfortunately, the survey does not provide data on rural areas.

**Table 1:** Results. Attitudes toward fairness and tax morale. Seventeen cities in Latin America. Multi-level, random intercept models

	Dependent variable:	
	Paying taxes is a civic duty	Evading taxes is unjustifiable
	(1)	(2)
Individual attitudes toward fairness and redistribution		
Taxes on rich people are low	− 0.249*** (0.057)	0.111** (0.054)
Merit > luck	0.268*** (0.061)	0.319*** (0.058)
Redistribution is a priority	− 0.084 (0.065)	− 0.115* (0.061)
Individual attitudes toward social contract		
Reciprocity	0.113*** (0.011)	− 0.024** (0.011)
Deterrence	0.030*** (0.011)	− 0.072*** (0.010)
Peer effects	0.112*** (0.014)	− 0.016 (0.013)
Individual socio-economic attributes		
Gender (male = 1)	0.277*** (0.058)	0.013 (0.055)
Age (from young to old)	0.013*** (0.002)	0.011*** (0.002)
Education (0 to postgraduate)	0.128*** (0.013)	0.087*** (0.012)
In the labor market (yes = 1)	0.052 (0.069)	− 0.011 (0.065)
Observations	7453	7459
Log Likelihood	− 16,911.320	− 16,519.230
Akaike Inf. Crit	33,848.640	33,064.460
Bayesian Inf. Crit	33,938.550	33,154.380

Data from Corporacion Andina de Fomento, CAF Survey 2011. *p < 0.1; **p < 0.05; ***p < 0.01

The results presented in Table 1 consistently show that individual attitudes toward fairness and merit are significantly associated with changes in tax morale. For example, estimates presented in model (1) suggest that individuals are more prone to believe that paying taxes is a civic duty if (i) they consider that taxes on rich people are not particularly low (i.e., the tax structure is relatively fair); and (ii) if they consider that merit is more significant than luck for personal economic success. The magnitude and statistical significance of these estimates are consistent across different model specifications and estimation techniques (e.g., see Table 3 in the Appendix). However, the estimate for my metric of preferences for redistribution—whether or not individuals think redistribution should be a top government priority—is not statistically significant.

Model (2) estimates these associations for individual attitudes toward tax evasion. In this case, the results suggest that individuals are more inclined to find tax evasion unjustifiable if (i) they think the tax structure is relatively unfair (taxes on rich people are too low) and (ii) merit is more significant than luck for economic progress. However, the estimate for the intensity of individual preferences for redistribution is not statistically significant.

Models (1) and (2) in Table 1 also evaluate the association of tax morale with reciprocity, deterrence, and peer effects. The evidence about reciprocity is sound. As suggested by other studies (Luttmer and Singhal 2014), individuals are more inclined to believe that paying taxes is a civic duty and that tax evasion is unjustifiable if they perceive that the government uses tax revenues to provide good-quality public services. Deterrence and peer effects reinforce the belief that paying taxes is a good citizen trait. Both estimates for deterrence and peer effects are positive and statistically significant in model (1). However, the empirical evidence on tax evasion is conflicting. Contrary to conventional expectations, model (2) estimates that a higher perception of deterrence makes tax evasion less—not more—unjustifiable, and peer effects seem to be statistically meaningless. This finding needs further investigation.

Finally, the empirical evidence presented in Table 1 also suggests that individual socioeconomic attributes are statistically associated with individuals’ tax morale. As expected, tax morale is higher among more educated and older individuals. Meanwhile, gender and labor market effects seem insignificant or inconsistent across different model specifications.

I assess the quality of these models using different tools. First, I use the *R* package *performance* to check the models’ predictors for collinearity. In both cases, the variance inflation factor is less than five for all the predictors, indicating a low correlation among them. Given the nature of our regression models, endogeneity could be a concern because any moderate correlation between some predictors and a random component or error term could significantly bias the coefficients and the variance components. Kim and Frees (2007) developed a technique for addressing endogeneity in multilevel models without needing external instrumental variables. In particular, I use their *multilevelIV()* function of the *R* package *REndo* to estimate my multilevel models specifying the regressors one can assume to be endogenous (e.g., taxing the rich). The *multilevelIV()* function returns the parameter estimates obtained with fixed effects, random effects, and the GMM estimator proposed by Kim and Frees (2007). Thus, we can make comparisons across models.

Following their testing protocol for higher-level endogeneity in multilevel settings, one would start by looking at the results of the omitted variable test comparing random effect estimators (REF) and fixed effects estimators at level two (FE-L2). If the null hypothesis is rejected, the model suffers from omitted variables. Then, one could test whether there are level-two omitted effects. To this end, one can rely on the model comparisons: fixed effects estimators at level two (FE-L2) versus GMM-L2 estimators. Table 4 in the Appendix presents the results of this test. In both cases, we can reject the null hypotheses that the models suffer from omitted variables bias, and consequently, one could argue that the level of endogeneity is low.

Thus far, the results support our hypothesis that a meaningful relationship exists between individual attitudes toward fairness and tax morale. Individuals seem less inclined to justify tax evasion if they consider taxes and access to economic opportunities relatively fair. I now examine specific ways that underpin this relationship. Does reciprocity reinforce the association between fairness and tax morale? To explore this hypothesis, I turn to assess possible mediation effects between “selfish” and “altruistic” considerations.

### Fairness, Reciprocity, and Tax Morale

**Fig. 1:**
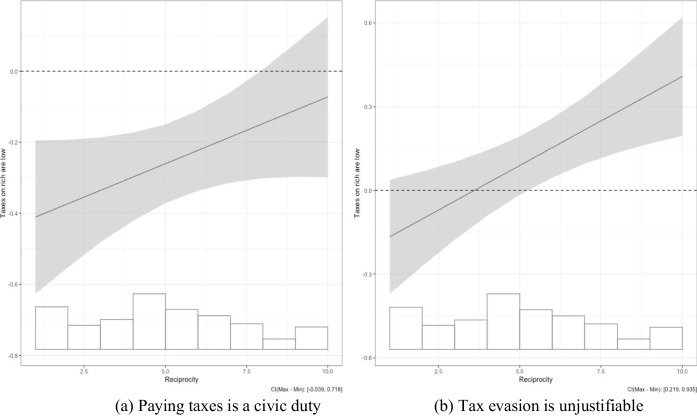
Mediation effects—tax fairness and reciprocity—contrast of linear predictions—based on models estimated in Table 5. **a** Paying taxes is a civic duty. **b** Tax evasion is unjustifiable

In the “Theory” section, I hypothesized that individuals’ views about fairness affect their views on tax morale only when their socioeconomic attributes or beliefs about reciprocity or meritocracy make them particularly sensitive to the drawbacks of inequality. We could expect tax morale to be low among individuals who believe that the tax structure is unfair or the government is not providing good quality public goods. To test this hypothesis, I estimate a series of hierarchical models of tax morale, but this time I include an interaction term between fairness and reciprocity. Figure 1 and Table 5 in the Appendix present the results.

As expected, individual beliefs about reciprocity attenuate the degree of association between fairness and tax morale (H2a). Panel (a) in Fig. 1 illustrates the relationship between individual views about fairness and tax morale at different levels of reciprocity. As shown in Table 1, individuals seem far less convinced that paying taxes is a trait of good citizens if they consider that taxes on rich people are low. However, panel (a) in Fig. 1 also shows that this negative association gradually disappears as reciprocity increases. The association between fairness and tax morale vanishes as taxpayers become more convinced that the government efficiently uses its revenues to provide good-quality public goods and services.

This interaction is even more solid for my metric of tax evasion. According to the results presented in panel (b) in Fig. 1, individuals increasingly agree that tax evasion is always unjustifiable when they are more concerned about tax fairness. This association is stronger when taxpayers believe the government uses its revenues to deliver good-quality public goods. However, at low levels of reciprocity, the association between fairness and individuals’ views about tax evasion is not statistically significant.

**Fig. 2:**
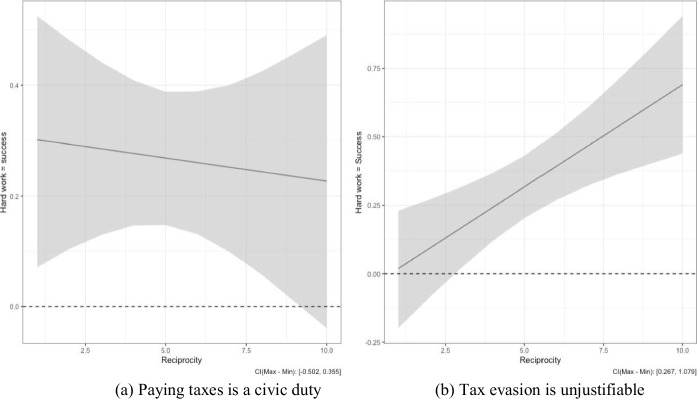
Mediation effects—merit and reciprocity—contrast of linear predictions—based on models estimated in Table 6. **a** Paying taxes is a civic duty. **b** Tax evasion is unjustifiable

**Fig. 3:**
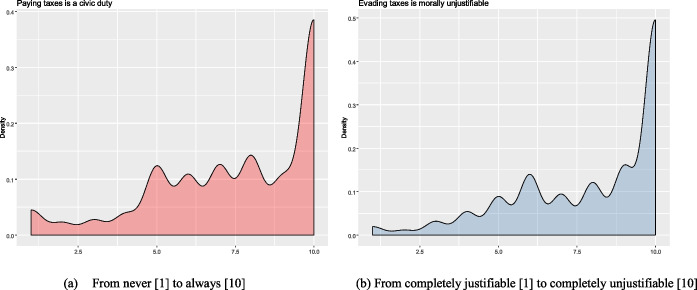
Density distributions, dependent variables. Source: ECAF Survey 2011

**Fig. 4:**
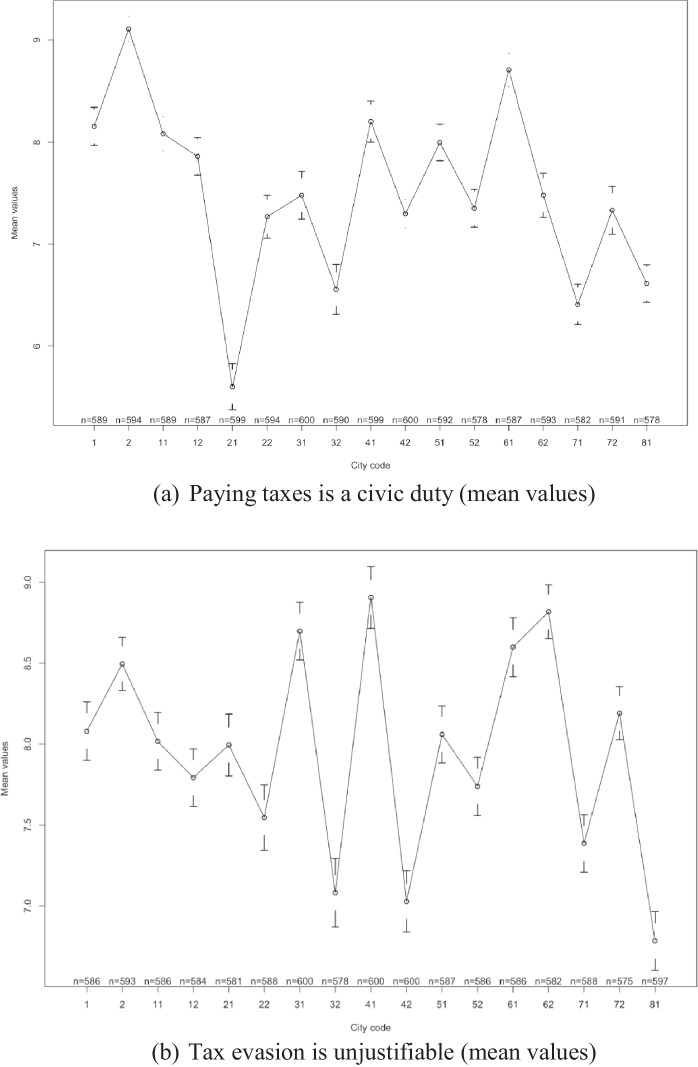
Mean values of the dependent variables by city. Source: ECAF Survey 2011

Does reciprocity also attenuate the association between merit and tax morale (H2b)? Fig. 2 and Table 6 in the Appendix show the relationship between personal views about merit and tax morale. As demonstrated in Table 1, individuals seem more convinced that paying taxes is a civic duty if they consider merit more significant than luck for personal economic success. Panel (a) in Fig. 2 shows that this association is independent of their views about reciprocity. No matter their beliefs about how efficient the government is in delivering public goods, individuals who believe that merit is more relevant than luck will always consider paying taxes as a trait of good citizens.

Reciprocity considerations boost the power of the relationship between merit and individuals’ views about tax evasion. Indeed, panel (b) in Fig. 2 shows that opinions about merit make individuals more likely to find tax evasion unjustifiable. This interaction is even more considerable when they believe the government uses its revenues to deliver good-quality public goods.

In summary, the empirical evidence presented above shows that fairness considerations are crucial to understanding citizens’ views about tax compliance. People’s views on tax fairness and merit shape their tax morale. However, I also show that these associations are not inelastic. They are sensitive to changes in people’s views about reciprocity. Individuals’ reciprocity concerns amplify the impact of fairness considerations on tax morale. As argued in the theory section, the empirical evidence presented here supports the idea that altruistic and selfish factors shape tax morale among individuals.

### Do We Get Similar Results Outside Latin America?

To partially examine the external validity of the results presented above, I run similar regression models using global rather than regional data—the sixth wave of the *World Values Survey* (*WVS6*) that puts together nationally representative surveys. It uses a standard questionnaire to assess beliefs and values among almost 85,000 respondents across more than 70 countries worldwide (Inglehart et al. 2014). In addition, this wave of surveys includes an item on tax evasion and several items assessing respondents’ attitudes toward fairness and redistribution. Some of these items are very similar to the ones I used in the previous statistical analysis. Therefore, one could run similar model specifications to evaluate whether or not the findings presented in the previous sections might be extrapolated to more extensive, global samples.

The *WVS6* questionnaire includes an item asking the respondents whether or not they consider cheating on their taxes justifiable. I use this question as a metric for tax morale—my primary dependent variable.

The *WVS6* also includes helpful questions to evaluate the strength of the relationship between attitudes toward fairness and tax morale. I use three items similar to those I employ in the models presented in Table 1. First, to assess individual attitudes toward tax fairness, I use an item asking respondents whether they think taxing the rich is an essential characteristic of democracy. Second, to assess people’s attitudes toward merit, I use an item asking the respondents whether they consider that hard work brings success or success is more a matter of luck and connections. Third, to assess the intensity of people’s preferences for redistribution, I use an item asking the respondents whether they think that incomes should be made equal or whether income differences are good incentives for individual effort. Finally, I also use other items from the survey to control for relevant personal socioeconomic attributes like age, gender, education, participation in the labor market, and social class.

Table 2 presents the results of a fixed-effects model estimation of tax morale among individuals across 75 countries. As predicted in the previous section (Table 1), individuals’ attitudes toward fairness are good predictors of their views on tax evasion. Individuals are less prone to justify tax evasion when considering that taxing the rich is essential for democracy. Similarly, tax morale is higher when individuals believe that merit is more significant than luck in bringing success.

**Table 2:** Results. Attitudes toward fairness and tax morale. 75 countries across the world. Fixed-effects models

	*Dependent variable:*
	*Cheating on Taxes is Justifiable*
Individual attitudes toward fairness and redistribution	
Taxing the rich is essential for democracy	− 0.009*** (0.002)
Merit is more important than luck	− 0.083*** (0.002)
Income should be made more equal	− 0.004** (0.002)
Individual socio-economic attributes	
In the labor market (yes = 1)	0.028** (0.012)
Social class – self-identification (from low to high)	0.041*** (0.007)
Gender (male = 1)	0.143*** (0.012)
Age (from younger to older)	− 0.011*** (0.0004)
Education (from none to postgraduate)	− 0.026*** (0.003)
Country fixed effects included	*Y*
Observations	*122,436*
R-squared	*0.586*
Adjusted R-squared	*0.586*
Residual Std. error	*2.020*
F Statistic	*2086.903*

Data from World Values Survey 2016. *p < 0.1; **p < 0.05; ***p < 0.01

Contrary to the models presented in Table 1, the *WVS6* metric of the intensity of preferences for redistribution is statistically significant. Furthermore, the results in Table 2 suggest that individuals who believe that society should equalize income distribution are also less inclined to justify cheating on taxes. Finally, the estimates for individual socioeconomic attributes are significant and go in the expected direction. For example, older and more educated individuals are less inclined to justify tax evasion. Meanwhile, individuals in the labor market and those who consider themselves members of higher social classes are more inclined to justify cheating on taxes.

The evidence presented in Table 2 also suggests that, even when using a larger sample of individual and geographical units, my main finding that fairness considerations are crucial to understanding citizens’ views about tax compliance remains statistically significant.

## Concluding Remarks

This paper focuses on the effect of individual attitudes toward fairness on people’s views about tax compliance. It shows that individuals who think the tax structure is unfair—i.e., it favors rich people—are less likely to consider paying taxes as a trait of good citizens and more likely to justify tax evasion. Moreover, this paper shows that negative views about tax fairness—e.g., if individuals believe that taxes on rich people are too low—are significantly associated with low morale among taxpayers. People are also less willing to pay taxes if they perceive that government does little to reduce inequality.

I also present empirical evidence that people’s views on reciprocity and meritocracy inform their opinions on tax compliance. My findings show that the relationship between attitudes toward fairness and tax evasion hinges critically on the heuristics people use to explain their position in income distribution. The more sensitive these views make people to inequality, the higher the effect of perceived fairness on their tax morale.

These findings help us understand the concept of reciprocity much better. I show that people perceive progressive tax schedules as reciprocal benefits. Furthermore, my findings suggest that tax morale increases when people think the tax structure is somewhat progressive. Therefore, this paper offers new empirical evidence to the literature on attitudes toward redistribution and progressive taxation. From this perspective, more than a better provision of public goods is needed to increase individual tax morale. Progressive tax schedules also reduce tax evasion because they improve people’s perception of fairness.

These findings might have significant policy consequences. If we accept that people’s views on redistribution make them more or less willing to pay their taxes, there are many tools that policymakers can use to make that link the basis of a more solid fiscal contract. On the one hand, my findings suggest that providing high-quality public goods and social policies to reduce inequality could significantly impact state capacity. Reducing inequality can create positive fiscal cycles that enhance fiscal capacity and improve governments’ position to lead economic change. On the other hand, policymakers—especially in developing countries—could make better efforts to make their distributional achievements more known among the population. If policymakers communicate better the impact of fiscal policies on the redistribution of income and assets, the public will be open to contributing more to building fiscal capacity—which is vital for economic development. In other words, making people more aware of the collective (and individual) benefits of fairness and tax justice could improve governments’ capacity to expand their fiscal space.

That is precisely why these findings are relevant beyond the case of Latin America. A better understanding of reciprocity is essential to assess the impact of people’s attitudes toward fairness on their tax preferences and willingness to pay taxes. However, it is also crucial to help governments establish a strong link between their social policy provision and fiscal capacity. My findings suggest that higher exposure to social benefits and information about tax progressivity could change citizens’ attitudes toward government action and make the fiscal contract more fluid. Furthermore, these findings suggest that increasing tax fairness could be an effective way to ensure fiscal stability and more sustainable and predictable sources of revenue. Last but not least, my findings support the emerging push for tax justice. Tax fairness is one of the most effective tools to reduce evasion, especially in contexts where other tools like deterrence and judicial institutions are fragile or contexts where labor market informality prevails, and governments face massive challenges to tax individuals or firms.

## Appendix

See Tables

**Table 3:** Results. Attitudes toward fairness and tax morale. Seventeen cities in Latin America. Fixed-effects models

	*Dependent variable:*	
	*Paying taxes is civic duty*	*Tax evasion is unjustifiable*
	*(1)*	*(2)*
Taxes on rich people are too low	− 0.250*** (0.057)	0.112** (0.054)
Merit is more important than luck	0.268*** (0.061)	0.318*** (0.058)
Redistribution should be a priority for government	− 0.084 (0.065)	− 0.116* (0.061)
Reciprocity	0.112*** (0.011)	− 0.025** (0.011)
Deterrence	0.030*** (0.011)	− 0.072*** (0.010)
Peer	0.113*** (0.014)	− 0.016 (0.013)
Gender (male = 1)	0.276*** (0.058)	0.012 (0.055)
Age (from young to old)	0.013*** (0.002)	0.011*** (0.002)
Education (from none to postgraduate)	0.128*** (0.013)	0.087*** (0.012)
In the labor market (yes = 1)	0.053 (0.069)	− 0.010 (0.065)
*Observations*	*7453*	*7459*
*R2*	*0.056*	*0.022*
*Adjusted R2*	*0.053*	*0.019*
*F Statistic*	*44.043***(df* = *10; 7426)*	*16.660***(df* = *10; 7432)*

Data from CAF Survey 2011. **p* < 0.1; ***p* < 0.05; ****p* < 0.01

^3^,

**Table 4:** Omitted variable tests for hierarchical models in Table 1 using REndo package

Model (1)			
	*df*	*Chisq*	*p value*
FE L2 VS REF	11	15.72	0.152
GMM L2 VS REF	11	19,806.09	< 2e-16***
Model (2)			
	*df*	*Chisq*	*p value*
FE L2 VS REF	11	16.45	0.125
GMM L2 VS REF	11	− 294,481.18	1.000

^∗^*p* < 0.1; ^∗∗^*p* < 0.05; ^∗∗∗^*p* < 0.01

^4^,

**Table 5:** Results. Attitudes toward fairness and reciprocity. Seventeen cities in Latin America. Mixed models including interaction

	*Dependent variable:*	
	*Paying taxes is civic duty*	*Tax evasion is unjustifiable*
	(1)	(2)
Taxes on rich people are too low	− 0.450*** (0.130)	− 0.233* (0.123)
Merit is more important than luck	0.270*** (0.061)	0.321*** (0.058)
Redistribution should be a government priority	− 0.084 (0.065)	− 0.116* (0.061)
Reciprocity	0.095*** (0.015)	− 0.054*** (0.015)
Tax rich*reciprocity	0.037* (0.022)	0.064*** (0.020)
Deterrence	0.029*** (0.011)	− 0.073*** (0.010)
Peer	0.112*** (0.014)	− 0.016 (0.013)
Gender (male = 1)	0.277*** (0.058)	0.013 (0.055)
Age (from young to old)	0.013*** (0.002)	0.011*** (0.002)
Education (from none to postgraduate)	0.128*** (0.013)	0.086*** (0.012)
In the labor market (yes = 1)	0.051 (0.069)	− 0.013 (0.065)
Constant	4.687*** (0.295)	7.562*** (0.253)
*Observations*	*7453*	*7459*
*Log Likelihood*	− *16,912.740*	− *16,517.350*
*Akaike Inf. Crit*	*33,853.470*	*33,062.690*
*Bayesian Inf. Crit*	*33,950.300*	*33,159.530*

Data from CAF Survey 2011. **p* < 0.1; ***p* < 0.05; ****p* < 0.01

^5^ and

**Table 6:** Results. Attitudes toward merit and reciprocity. Seventeen cities in Latin America. Mixed models including interaction

	*Dependent variable:*	
	*Paying taxes is civic duty*	*Tax evasion is unjustifiable*
	(1)	(2)
Taxes on rich people are too low	− 0.249*** (0.057)	0.110** (0.054)
Merit is more important than luck	0.311** (0.137)	− 0.055 (0.130)
Redistribution should be a priority for the government	− 0.084 (0.065)	− 0.113* (0.061)
Reciprocity	0.119*** (0.021)	− 0.078*** (0.020)
Merit*Reciprocity	− 0.009 (0.025)	0.074*** (0.023)
Deterrence	0.030*** (0.011)	− 0.072*** (0.010)
Peer	0.112*** (0.014)	− 0.016 (0.013)
Gender (male = 1)	0.277*** (0.058)	0.014 (0.055)
Age (from young to old)	0.013*** (0.002)	0.011*** (0.002)
Education (from none to postgraduate)	0.128*** (0.013)	0.087*** (0.012)
In the labor market (yes = 1)	0.052 (0.069)	− 0.012 (0.065)
Constant	4.546*** (0.300)	7.637*** (0.259)
*Observations*	*7453*	*7459*
*Log Likelihood*	− *16,914.050*	− *16,516.930*
*Akaike Inf. Crit*	*33,856.090*	*33,061.870*
*Bayesian Inf. Crit*	*33,952.920*	*33,158.710*

Data from CAF Survey 2011. **p* < 0.1; ***p* < 0.05; ****p* < 0.01

^6^.

See Figs. ^3^ and ^4^.
